# COVID-19 vaccination hesitancy for children: A pilot assessment of parents in the United States

**DOI:** 10.34172/hpp.2022.51

**Published:** 2022-12-31

**Authors:** Kavita Batra, Manoj Sharma, Chia-Liang Dai, Ravi Batra, Jagdish Khubchandani

**Affiliations:** ^1^Department of Medical Education, Kirk Kerkorian School of Medicine at UNLV, University of Nevada, Las Vegas, USA; ^2^Department of Social & Behavioral Health, School of Public Health, University of Nevada, Las Vegas, USA; ^3^Department of Internal Medicine, Kirk Kerkorian School of Medicine at UNLV, University of Nevada, Las Vegas, USA; ^4^Department of Teaching and Learning, College of Education, University of Nevada, Las Vegas, USA; ^5^Department of Public Health Sciences, New Mexico State University, New Mexico, USA

**Keywords:** Coronavirus disease 2019, COVID-19 vaccine, Children, Prevention, Public health, Vaccine confidence, Vaccine literacy

## Abstract

**Background:** Coronavirus disease 2019 (COVID-19) vaccine hesitancy has remained a significant concern among adults worldwide. However, not much is known about parental vaccine hesitancy for getting children vaccinated for COVID-19 in the U.S. Thus, the purpose of this study was to conduct a national assessment of parents’ preferences for COVID-19 vaccination of children using the evidence-based Multi-Theory Model (MTM) and explore the predictors of vaccine hesitancy.

**Methods:** To participate in this study, a national random sample of parents (n=263) took a valid and reliable online questionnaire based on the MTM. Independent samples *t* test, chi-square test, multiple logistic regression was utilized to analyze data.

**Results:** More than two-fifths (42%) of the participating parents were not willing to get their children vaccinated for COVID-19. Parental vaccination status, booster dose acceptance, education, and political affiliation were significant predictors of willingness to get children vaccinated for COVID-19. In the multiple logistic regression analyses, behavioral confidence and participatory dialogue (i.e., perceived advantages versus disadvantages) were statistically significant predictors of COVID-19 vaccination hesitancy for children among the participating parents.

**Conclusion:** Given the multiple factors that were found influential in parental hesitancy for COVID-19 vaccination among children, multimodal and evidence-based interventions are needed to increase the uptake of COVID-19 vaccines among children by influencing the parents’ perceptions, increasing their confidence, dispelling misinformation, and reducing constraints for vaccination. Such interventions should emphasize communication and messaging that is truthful, interactive, scientifically correct, and to be delivered in a variety of community-based settings.

## Introduction

 Coronavirus disease 2019 (COVID-19) infections continue to disrupt lives and economies around the world. Vaccines for the prevention of COVID-19 have been available since late 2020 and early 2021. Despite the availability of COVID-19 vaccines and in many parts of the world now, a large proportion of the global population remains unvaccinated. In part, this is because of vaccine hesitancy and refusal.^1-5^ A plethora of studies worldwide has explored the extent and predictors for COVID-19 vaccine hesitancy among adults. These studies also highlight the reasons for widespread COVID-19 vaccination hesitancy.^4-8^ For example, a global review during the early phases of the vaccine rollout found that the rate of COVID-19 acceptance was 23.6% to 97% across 33 countries.^6^ In contrast, a review of data till February 2021 from 82 studies found that global vaccine hesitancy rates were 10–57.8% and vaccine refusal rates were in the range of 0–24%.^4^ Subsequently, a June 2021 study of 23 000 participants from 23 countries found that the average rate of vaccine acceptance was 75.2%.^7^ In the United States (U.S.), before the vaccine rollout in late 2020, more than a fifth of the adults did not want to take the COVID-19 vaccine. In alignment with these findings, the latest estimates from July 2022 suggest that almost a fourth of American adults remain either partially vaccinated or unvaccinated.^1,2,8^ The most common reasons found in global studies for not obtaining COVID-19 vaccination are fear of side effects, lack of trust in vaccines or authorities, misinformation and myths, low perceived risk of COVID-19 infections, and religious or cultural beliefs.^2,4,6,7^

 Within a few months of the COVID-19 vaccine rollout for adults (in late 2020), many countries started administering the vaccines to children.^9-12^ For example, the United States Food and Drug Administration (FDA) approved the use of COVID-19 vaccines for children 12-15 years of age by the summer of 2021.^9^ Around the same time or quickly in succession, the European Union and other countries ap-proved the use of vaccines for children of this age group.^10,11^ By late 2021, COVID-19 vaccination approvals were expanded to include children 5-11 years old.^9,11^ More recently, by the summer of 2022, these vaccines have also been approved for use among children 6 months to 5 years old.^9^ Similar to vaccine hesitancy among adults, a few studies have discussed COVID-19 vaccination hesitancy among parents to get their children vaccinated.^12-16^

 One of the earliest studies on parental COVID-19 vaccine hesitancy in early 2021 found that higher education of parents was associated with greater willingness to obtain COVID-19 vaccines for themselves and their children.^12^ Subsequent studies from various parts of the world found that half or more of the parents were either un-sure or not willing to get their children vaccinated for COVID-19.^13-15^ The major reasons discussed in these studies for COVID-19 vaccine hesitancy among parents (to get children vaccinated) were lower income/education or perceived risk of infection among children, parental vaccine hesitancy; concerns about vaccine safety and side effects, belief that vaccines can cause infection or transmit the infection to adults, dependence on social media for information, etc.^13-16^ Having children at risk of COVID-19 or with chronic conditions, knowing others who got vaccinated, a belief that vaccines have been tested extensively and are safe and effective, and recommend-dations from healthcare providers were associated with a higher willingness to get children vaccinated with the vaccines.^14-17^

 The extant literature on parental COVID-19 vaccination hesitancy has certain limitations.^12-20^ For example, most studies are from outside the U.S., have small sample sizes, use convenience/ snowball sampling, do not adequately quantify parental COVID-19 vaccine hesitancy, and assess a limited number of variables associated with vaccine hesitancy. Above all, in our review of studies on parental COVID-19 vaccination hesitancy, we could not find assessments that use evidence-based behavioral intention theories and behavior change models. Thus, the purpose of this investigation was to comprehensively assess the American parents’ COVID-19 vaccination hesitancy in getting their children vaccinated using the Multi-Theory Model (MTM) of health behavior change. The MTM was designed to address both initiation and sustenance of health behavior change but, in this study, only the initiation model was operationalized that entails the constructs of participatory dialogue (in which advantages of behavior change outweigh the disadvantages), behavioral confidence, and changes in the physical environment.^1-5^ This theory integrates cognitive, conative, and environmental factors that are intended to be utilized for designing health behavior change interventions.^1,3,5,6^ MTM constructs are adaptable across health behaviors and have been used to explain a variety of health behaviors (including an explanation of COVID-19 vaccination hesitancy in adults).^1,3,5^

## Material and Methods

###  Study design and Sample

 This cross-sectional analytical study was conducted in October 2021 and utilized a web-based survey to recruit a diverse sample of U.S. adults. Qualtrics Research Marketing Team was contracted to manage the data collection and for participant recruitment. Participants were provided incentives per terms and conditions set forth by Qualtrics and its data collection partners. Respondents were invited through emails, in-app notifications, and specialized campaigns for relatively inaccessible population subgroups. A non-probability sampling method was utilized for the data collection until the desired quota constraints are met. The sample requirement was pre-estimated using the formula: n = (z)2 p (1 − p)/d2 considering a 90% confidence interval, a margin of error d = 5%, and the proportion of parental hesitancy to get their children vaccinated was 28% based on the recent data reported by Yadete and colleagues.^20^ Based on the aforementioned inputs, the minimum sample required was 243 (221 + 10% non-response = 243) after factoring in 10% non-response. This study received exempt status from the Institutional Review Board (or Ethics Committee) of the University of Nevada, Las Vegas (UNLV-2021-108).

###  Variables and measures

 The survey instrument consisted of 52 items related to vaccine confidence, vaccine literacy, multilevel-theory-model-based initiation of vaccination behavior, and demographic questions.^1,3,19-22^ The MTM survey instrument has been validated by a previous study,^5^ which established construct validity of the MTM subscales through the confirmatory factor analysis with maximum likelihood estimation. The referenced study found a single-factor solution with all factor loadings over the threshold of 0.32. In addition, Eigenvalue criteria was satisfied in all the MTM subscales with value greater than one. Reliability diagnostics revealed appropriate Cronbach alpha value of at least 70% in all the MTM subscales.

 Vaccine literacy (i.e., functional, interactive, and critical) includes knowledge and competence to deal with information concerning vaccines, and the vaccine confidence index measures the propensity toward vaccines (this measure has been developed for the influenza vaccine and was adapted in the present study for COVID-19 vaccination).^19-22^

 A set of questions related to the parents’ vaccination status were also asked, which allowed us to investigate the association between parental willingness to accept the vaccine and their hesitancy towards getting their children vaccinated.^14-17,19-22^ The dependent variable used in this study was the parental willingness to get their children vaccinated, which was dichotomized into “yes” and “no” categories. Independent variables included demographic characteristics, primary series vaccination status, and booster-dose acceptability of the parents. Additionally, information related to the source of the COVID-19 vaccine information, and the level of trust in the informational sources were also measured.

###  Statistical analysis

 Means and standard deviation were used to present continuous variables, where-as categorical variables were represented as counts and percentages. Bivariate (e.g., chi-square, independent samples *t *test, and Pearson’s correlation) and multiple logistic regression tests were used to analyze the data. For the logistic regression, we used the maximum likelihood method to obtain Wald’s confidence intervals and adjusted odds ratio estimates. All variables were dummy coded to allow an appropriate estimation in the regression, and probability in the logistic regression was modeled on “willingness of parents” = “yes.” Statistical significance was assumed at *P* < 0.05. All data analyses were conducted using the Statistical Package for Social Sciences (SPSS) version 27 for Windows (Armonk, NY, USA: IBM Corp) and Statistical Analysis System (SAS), version 9.4 (SAS Institute, Cary, NC).

## Results

 Among the total of 263 participating parents, most (58.2%, 95% CI: 51.9%, 64.2%) were willing to get their children vaccinated and the remaining (41.8%, 95% CI: 35.8%, 48.0%) were hesitant (Table 1). Among parents who were willing, a significantly larger proportion was fully vaccinated (88.9% vs. 45.5%, *P* < 0.001), had greater acceptability towards booster dose (64.1% vs. 29.1%, *P* < 0.001), had a graduate degree (13.7% vs. 5.5%, *P* = 0.04), and had a democratic political affiliation (50.3% vs. 22.7%) as opposed to their hesitant counterparts (Table 1). No significant differences were found by age, gender, race/ethnicity, marital status, income, region, urbanity, and religious affiliations.

**Table 1:** Demographic characteristics and bivariate comparisons of the parents’ respondents (N = 263)

**Variable name**	**Categories**	**Overall ** **No. (%)**	**Willingness to have children vaccinated **		***P*** ** value**
			**Yes, 153 (58.2)**	**No, 110 (41.8)**	
Vaccinated status (primary series) of the parents	Yes	186 (70.7)	136 (88.9)	50 (45.5)	< 0.001
	No	77 (29.3)	17 (11.1)	60 (54.5)	
Hesitancy of parents toward COVID-19 booster dose	Yes	133 (50.6)	55 (35.9)	78 (70.9)	< 0.001
	No	130 (49.4)	98 (64.1)	32 (29.1)	
Age groups	18-44 years	150 (57.0)	87 (56.9)	63 (57.3)	0.212
	45-64 years	61 (23.2)	31 (20.3)	30 (27.3)	
	65 years or older	52 (19.8)	35 (22.9)	17 (15.5)	
Gender	Male	123 (46.8)	73 (48.0)	50 (46.7)	0.801
	Female	136 (51.7)	79 (52.0)	57 (53.3)	
Race/ethnicity	Non-Hispanic White	129 (58.1)	75 (49.0	54 (49.1)	0.310
	Non-Hispanic African American	42 (16.0)	29 (19.0)	13 (11.8)	
	Hispanic	72 (27.4)	40 (26.1)	32 (29.1)	
	Other (including multiracial groups)	20 (7.6)	9 (5.9)	11 (10.0)	
Marital status	Divorced/Separated	26 (9.9)	17 (11.1)	9 (8.2)	0.321
	Other	10 (3.8)	3 (2.0)	7 (6.4)	
	Single, never married	81 (30.8)	49 (32.0)	32 (29.1)	
	Married	128 (48.7)	75 (49.0)	53 (48.2)	
Education	High school diploma or GED	79 (30.0)	50 (32.7)	29 (26.4)	0.040
	4-year college degree	57 (21.7)	34 (22.2)	23 (20.9)	
	Graduate level degree*	27 (10.3)	21 (13.7)	6 (5.5)	
	Some high school	10 (3.8)	4 (2.6)	6 (5.5.)	
	Other, including some college/vocational training schools*	90 (34.2)	44 (28.8)	46 (41.8)	
Income	$10,000 or below	22 (8.4)	9 (6.3)	13 (13.1)	0.414
	$10,001-$25,000	39 (14.8)	22 (15.5)	17 (17.2)	
	$25,001-$50,000	73 (27.8)	45 (31.7)	28 (28.3)	
	$50,001-$100,000	84 (31.9)	52 (36.6)	32 (32.3)	
	Above $100,001	23 (8.7)	14 (9.9)	9 (9.1)	
Region	Midwest	48 (18.3)	26 (17.0)	22 (20.0)	0.901
	Northeast	53 (20.2)	31 (20.3)	22 (20.0)	
	South	125 (47.5)	74 (48.4)	51 (46.4)	
	West	37 (14.1)	22 (14.4)	15 (13.6)	
Urbanity	Rural	66 (25.1)	34 (22.2)	32 (29.1)	0.411
	Suburban	106 (40.3)	66 (43.1)	40 (36.4)	
	Urban	91 (34.6)	53 (34.6)	38 (34.5)	
Political affiliation	Democrat	102 (38.8)	77 (50.3)	25 (22.7)	< 0.001
	Republican	68 (25.8)	34 (22.2)	34 (30.9)	
	Independent including other*	93 (35.4)	42 (27.5)	51 (46.4)	
Religion	Christianity	155 (58.9)	93 (60.8)	62 (56.4)	0.721
	Religiously unaffiliated	33 (12.5)	19 (12.4)	14 (12.7)	
	Others	75 (28.5)	41 (26.8)	34 (30.9)	

*Note:* Some percentages may not add up to 100% as a few respondents preferred not to answer. Other religions include Hinduism, Judaism, Buddhism, Islam, etc.; Categories marked with an asterisk had adjusted residuals greater than 2 and were significant; GED: General Educational Development; COVID-19: Coronavirus Disease 2019; Bolded *P* values are statistically significant.

 The mean scores of vaccine literacy (42.62 ± 7.04 vs. 39.86 ± 7.00, p = 0.002) and vac-cine confidence index (2.23 ± 1.24 vs. 1.06 ± 0.68, *P* < 0.001) were statistically significantly higher among parents who were willing to get their children vaccinated as opposed to those who were not willing (Table 2).

**Table 2:** Vaccine literacy and confidence among parents based on vaccine hesitancy (N = 263)

**Variable name**	**Willingness to have children vaccinated**		***P*** ** value**
	**Yes (n=153)**	**No (n=110)**	
Functional literacy	14.48 ± 4.41	13.76 ± 3.82	0.201
Interactive literacy	15.36 ± 3.39	14.34 ± 3.56	0.020
Critical literacy	12.77 ± 2.96	11.75 ± 2.93	0.006
Total vaccine literacy	42.62 ± 7.04	39.86 ± 7.00	0.002
Vaccine Confidence Index	2.23 ± 1.24	1.06 ± 0.68	< 0.001

Note: All measures are represented as Mean ± standard deviation unless stated otherwise; Bolded *P* values are statistically significant.

 For mean the scores of parental perceived advantages towards vaccines, behavioral confidence, and changes in the physical environment, there were statistically significant differences among those willing vs. non-willing to get their children vaccinated (Table 3).

**Table 3:** MTM initiation and its subscale scores among parents based on vaccine hesitancy (N = 263)

**MTM construct**	**Willingness to have children vaccinated**		***P*** ** value**
	**Yes (n=153)**	**No (n=110)**	
Overall initiation score (Possible score:0-4)	3.14 ± 1.13	1.12 ± 1.28	< 0.001
Subscales			
Perceived advantages(Possible score: 0-12)	8.92 ± 2.67	4.28 ± 3.16	< 0.001
Perceived disadvantages(Possible score: 0-12)	5.25 ± 3.27	7.61 ± 3.27	< 0.001
Participatory dialogue* (Possible score: -12 to + 12)	3.46 ± 1.30	1.46 ± 0.86	< 0.001
Behavior confidence (Possible score: 0-12)	8.75 ± 3.15	4.07 ± 3.50	< 0.001
Changes in the physical environment (Possible score: 0-12)	8.97 ± 3.01	4.93 ± 3.90	< 0.001

*Note:* All measures are represented as Mean ± standard deviation unless stated otherwise; Participatory dialogue was transformed to aid interpretability; MTM: Multi-theory Model; Bolded *P* values are statistically significant.

 Bivariate analysis between the source of information and willingness of parents revealed that a significantly higher proportion of willing parents relied on public health organizations for COVID-19 information compared to parents who were not willing to get their children vaccinated (26.1% vs. 14.5%, *P* = 0.02, Table 4). Compared to the willing, a significantly larger proportion of non-willing parents had ‘a very little’ (12.4% vs. 25.5%, *P* = 0.01) or ‘no trust at all’ (2.6% vs. 20.9%, *P* < 0.001) in the informational source for vaccines (Table 4).

**Table 4:** Sources of information and degree of trust in COVID-19-related information

**Variable name**	**Categories**	**Overall ** **No. (%)**	**Willingness to have children vaccinated **		***P*** ** value**
			**Yes, 153 (58.2)**	**No, 110 (41.8)**	
Source of COVID-19 vaccine information	Friends/Family	31 (11.8)	18 (11.8)	13 (11.8)	0.912
	Healthcare provider	60 (22.8)	34 (22.2)	26 (23.6)	0.813
	Public health organizations	56 (21.3)	40 (26.1)	16 (14.5)	0.020
	Social media	31 (11.8)	15 (9.8)	16 (14.5)	0.211
	Television (including national and local channels)	56 (21.3)	36 (23.5)	20 (18.2)	0.308
	Others, including radio and newspaper	29 (11.0)	10 (6.5)	19 (17.3)	0.010
Trust in the informational source	A lot	93 (35.4)	80 (52.3)	13 (11.8)	< 0.001
	Somewhat	96 (36.5)	50 (32.7)	46 (41.8)	0.131
	Very little	47 (17.9)	19 (12.4)	28 (25.5)	0.010
	Not at all	27 (10.3)	4 (2.6)	23 (20.9)	< 0.001

COVID-19, Coronavirus Disease 2019; Bolded *P* values are statistically significant.

 In a bivariate correlation test, a significant and positive correlation was noted between “perceived advantages” and “behavioral confidence” (*r* = 0.703, *P* < 0.001); “changes in the physical environment” and “behavioral confidence” (*r* = 0.797, *P* < 0.001), interactive vaccine literacy and critical literacy (*r* = 0.724, *P* < 0.001), and total vaccine literacy with interactive literacy (*r* = 0.747, *P* < 0.001; Table 5). The correlation between vaccine confidence and “perceived advantages” was also positive and statistically significant (r = 0.627, *P* < 0.001).

**Table 5:** Pearson correlations, and reliability estimates for study variables in the sample of parents (n = 263)

**Variable**s	**1**	**2**	**3**	**4**	**5**	**6**	**7**	**8**	**9**	**10**
1. Perceived advantages	1	-0.330**	0.703**	0.658**	0.101	0.253**	0.055	0.219**	0.272**	0.627**
2. Perceived disadvantages	-0.330**	1	-0.318**	-0.277**	-0.162**	-0.175**	-0.368**	0.041	0.048	-0.623**
3. Behavioral confidence	0.703**	-0.318**	1	0.797**	0.125*	0.338**	0.166**	0.266**	0.266**	0.579**
4. Change in the physical environment	0.658**	-0.277**	0.797**	1	0.220**	0.394**	0.150*	0.355**	0.315**	0.548**
5. Age	0.101	-0.162**	0.125*	0.220**	1	0.191**	0.233**	0.001	0.131*	0.315**
6. Total literacy score	0.253**	-0.175**	0.338**	0.394**	0.191**	1	0.539**	0.747**	0.762**	0.389**
7. Functional literacy	0.055	-0.368**	0.166**	0.150*	0.233**	0.539**	1	-0.078	-0.019	0.379**
8. Interactive literacy	0.219**	0.041	0.266**	0.355**	0.001	0.747**	-0.078	1	0.724**	0.155*
9. Critical literacy	0.272**	0.048	0.266**	0.315**	0.131*	0.762**	-0.019	0.724**	1	0.218**
10. Vaccine confidence	0.627**	-0.623**	0.579**	0.548**	0.315**	0.389**	0.379**	0.155*	0.218**	1

**P < .01; *P < 0.05; 1: Perceived advantages; 2: Perceived disadvantages; 3: Behavioral confidence; 4: Change in the physical environment; 5: Age; 6: Total literacy score; 7: Functional literacy; 8. Interactive literacy; 9. Critical literacy; 10. Vaccine confidence

 The results of logistic regression (Table 6) indicated that parental vaccination status, behavioral confidence, and participatory dialogue were the statistically significant predictors of parents’ willingness to get their children vaccinated. With the increase in behavioral confidence and participatory dialogue, the odds of parental willingness to get their children vaccinated increased by 1.27 (AOR = 1.277, *P =*0.01) and 1.22 times (AOR = 1.229, *P =*0.008) respectively. Parents who were fully vaccinated has nearly 3.5 times greater odds in favor of their children getting the COVID-19 vaccine than parents who were not fully vaccinated (*P =*0.01).

**Table 6:** Multiple logistic regression investigating predictors of parents’ willingness to have their children vaccinated (N = 263)

**Variabl**e	**Odds ratio**	**95% Confidence limits**		***P*** ** value**
Age	0.990	0.961	1.019	0.501
Gender, male vs. female	0.513	0.215	1.224	0.110
Race/Ethnicity, Black vs. White	0.520	0.154	1.754	0.301
Hispanic vs. White	0.344	0.107	1.102	0.070
Another race vs. White	0.739	0.127	4.279	0.712
Married vs. divorced/separated	0.679	0.144	3.206	0.611
Single vs. divorced/separated	1.662	0.341	8.107	0.517
Other vs. divorced/separated	0.309	0.042	2.300	0.321
Republican vs. Democrats	0.397	0.132	1.191	0.090
Other, including independent vs Democrats	0.590	0.227	1.533	0.312
Christianity vs. religiously unaffiliated	0.759	0.204	2.829	0.710
Other religion vs. religiously unaffiliated	0.329	0.078	1.389	0.111
West vs. South	1.691	0.360	7.933	0.510
Northeast vs. South	0.400	0.136	1.178	0.091
Midwest vs. South	0.983	0.326	2.961	0.912
Other (Some college/Vocational schools) vs. some high school	0.791	0.103	6.064	0.871
High school diploma or GED vs. some high school	1.490	0.208	10.678	0.715
4 years of college degree vs. some high school	0.938	0.112	7.836	0.912
Graduate vs. Some high school	1.346	0.130	13.913	0.821
Income, $10.001-$25,000 vs. income under $10,000	0.833	0.201	3.453	0.821
Income, $25,001-$50,000 vs. income under $10,000	2.450	0.663	9.055	0.215
Income, $50,001-$100,000 vs. income under $10,000	1.699	0.428	6.750	0.512
Income, above $100,000 vs. income under $10,000	1.573	0.280	8.836	0.621
Suburban vs. rural	1.493	0.504	4.428	0.514
Urban vs. rural	1.489	0.513	4.323	0.521
Trust on information source, A lot vs. not at all	1.966	0.266	14.564	0.511
Trust on information source, somewhat vs. not at all	0.925	0.145	5.918	0.920
Trust on information source, very little vs. not at all	2.061	0.314	13.514	0.513
Primarily vaccinated (parents), Yes vs. No	3.471	1.339	8.997	0.010
Vaccine confidence index	1.262	0.622	2.560	0.511
Vaccine literacy	0.997	0.926	1.073	0.901
Participatory dialogue	1.229	1.056	1.430	0.008
Behavioral confidence	1.277	1.053	1.548	0.011
Changes in the physical environment	1.054	0.887	1.254	0.511

GED: General Educational Development; Bolded *P* values are statistically significant.

## Discussion

 In this national study, we identified the correlates of parents’ willingness to have their children vaccinated with the COVID-19 vaccine based on the initiation con-structs of MTM, vaccine literacy, vaccine confidence index, sources of information, and trust of informational sources. The majority of the parents (58.2%) of the parents were willing to get their children vaccinated. Parents who were fully vaccinated or did not have hesitancy toward getting COVID-19 vaccine boosters had a higher willing-ness to have their children vaccinated. These findings have been confirmed in other studies outside of the U.S.^14,15,17^ Furthermore, parents with higher education or Democratic political affiliation were more likely to have their children vaccinated. Education and Democrat political affiliation are strong predictors for adult COVID-19 vaccination preferences and also seem to hold an influence on the tendency to get children vaccinated.^1-3,23-25^ Interventions geared towards parents with lesser education are warranted to ensure robust uptake of COVID-19 vaccination among children.^12-14^

 With regard to MTM constructs, scores of constructs (i.e. participatory dialogue, behavioral confidence, and physical environment changes) were significantly higher among those willing to have their children COVID-19 vaccinated. Furthermore, behavioral confidence and participatory dialogue along with parental self-status of getting vaccinated were significant predictors of parents’ willingness to get their children vaccinated with the COVID-19 vaccine. This model accounted for 66.6% of the variance in explaining the willingness of parents to get the COVID-19 vaccine for their children. These findings are consistent with other studies of adults examining this model in the context of COVID-19 vaccination.^1,3,5,19^ Interventions to increase parents’ confidence, perceptions of vaccine advantage, and COVID-19 infection-related risks should be implemented in community-based settings and doctors’ office.^13,14,16,20,24,25^ In addition, trust building will play a vital role in improving the vaccine uptake. There are some historical concerns why people distrust healthcare systems in the U.S. and also among other countries.^26^ Asadi and Colleagues investigated general attitudes of people residing in African and Middle East countries towards the COVID-19 vaccine and cited “mistrust” as an important predictor of vaccine hesitancy.^26^

 For the trust building, a diverse, multisector set of partners and collaborators (collective responsibility) at global, national, and local levels will be instrumental.^27^ All dimensions of trust, including trust in the quality and safety of vaccines, institutional trust, and inter-personal trust in the professionals, who are involved in the communication and vaccines administration, will require a multipronged approach in the trust building process.^27^ Clear and effective messaging through community leaders, healthcare providers, public health professionals, and employers will be essential.

 Vaccine literacy played a significant role in determining parents’ willingness to have their children vaccinated with the COVID-19 vaccine. Our findings showed that parents who were willing to have their children vaccinated performed significantly higher on total vaccine literacy than those who were not willing to have their children vaccinated. A recent study found that a substantial proportion of U.S. adults had very low coronavirus-related literacy scores; highlighting the need for greater health literacy.^28^ Similar to health literacy, vaccine literacy not only indicates the acquired knowledge of vaccines but also involves a person’s competence to locate credible in-formation and make responsible decisions about the vaccination. Increasing vaccine literacy should be a focus for future COVID-19 prevention efforts for children as well as adults.^1-3,12-14^ Likewise, we found that parents who were willing to have their children vaccinated had greater confidence in vaccines compared to hesitant parents. To increase COVID-19 vaccine confidence, parents’ concerns regarding vaccines should be addressed and communication with parents should emphasize conveying evidence-based information about vaccine safety, efficacy, and the development process.^12,13,17,28,29^ We also found that if the COVID-19 vaccine-related sources of information were public health organizations, the parents were more likely to get their children vaccinated. Interestingly, and in contrast, messaging from radio and news-papers led to more parents being reluctant toward getting their children vaccinated. Trust in the informational source also played a significant role for parents willing to get their children vaccinated. Public health organizations need to play a key role in disseminating evidence-based and truthful information to help increase COVID-19 vaccination among children.^27-29^ Finally, the construct of behavioral confidence can be built by helping people overcome the barriers to vaccination. This would also relate to the construct of changes in the physical environment wherein the issues of accessibility and affordability of COVID-19 vaccines especially for high-risk groups would need to be addressed.^1-6,14-19,24^

###  Strengths and limitations

 To authors’ best knowledge, this study is the first one to perform theory-based assessment of the parental hesitancy in getting their children vaccinated. However, this study is not without limitations. First, cross-sectional nature of this study limited our ability to establish causality. Second, certain type of biases (e.g., selection, recall, and non-response bias) were inevitable. Also, some parents may have provided socially desirable responses, which might have introduced social desirability bias. Third, there could be individual characteristics and other influential factors that might have left unmeasured and could have influenced study participants’ willingness to get their children vaccinated (e.g., side effects of vaccines among parents). Next, as the sample is limited in nature and extent (e.g., limited to those with computers or mobile phones and an understanding of the online survey environment), external validity will be limited. Moreover, sample was not representative, which might limit the external validity of the results. Future studies can be planned with a nationally representative sample to investigate parental hesitancy.

## Conclusion

 This study purported to examine the correlates of parents’ willingness to have their children vaccinated with the COVID-19 vaccine. Nearly two in five parents were not willing to get their children vaccinated and education, political affiliation, or pa-rental vaccination status were significant predictors of parents’ willingness to get children vaccinated for COVID-19. Also, the key constructs of MTM (i.e., vaccine literacy, vaccine confidence, trust in information sources, participatory dialogue, behavioral confidence, and physical environment) were influential in predicting parents’ willingness to have their children receive COVID-19 vaccination. Given that multiple factors predict parental hesitancy for COVID-19 vaccination among children, multi-modal interventions need to be implemented in healthcare provider offices and community settings with a focus on communication, confidence building, constraint removal, calculation in favor of vaccines, and collective responsibility to prevent the spread of COVID-19 infections.

## Acknowledgements

 Authors would like to thank their respective institutions for their kind support.

## Author Contributions

**Conceptualization:** Kavita Batra and Manoj Sharma.

**Methodology:** Manoj Sharma, and Kavita Batra.

**Software: **Kavita Batra, Chia-Liang Dai.

**Validation:** Manoj Sharma, Kavita Batra, Chia-Liang Dai, Ravi Batra and Jagdish Khubhchandani.

**Formal analysis:** Kavita Batra, and Ravi Batra.

**Investigation:** Manoj Sharma, Kavita Batra, Ravi Batra, Chia-Liang Dai, Jagdish Khubhchandani.

**Resources:** Manoj Sharma.

**Data curation:** Manoj Sharma, and Kavita Batra.

**Writing—original draft:** Manoj Sharma, Kavita Batra, Chia-Liang Dai, Ravi Batra, Jagdish Khubhchandani.

**Writing—review and editing:** Manoj Sharma, Kavita Batra, Chia-Liang Dai, Ravi Batra, Jagdish Khubhchandani.

**Visualization:** Kavita Batra, and Ravi Batra.

**Supervision:** Manoj Sharma and Kavita Batra.

**Project administration:** Manoj Sharma and Kavita Batra.

**Funding acquisition:** Manoj Sharma.

 All authors have read and agreed to the published version of the manuscript.

## Funding

 This research study was funded by the School of Public Health, University of Nevada, Las Vegas, internal grant number PG03008.

## Ethical Approval

 The study was conducted according to the guidelines of the Declaration of Helsinki, and approved by the Institutional Review Board (or Ethics Committee) of the University of Nevada, Las Vegas (UNLV-2021-108 dated 4 October 2021). All research activities were performed in accordance with the Declaration of Helsinki. Informed consent was obtained from all subjects involved in the study.

## Competing Interests

 None.
